# Methodological aspects to be considered when measuring the approximate number system (ANS) – a research review

**DOI:** 10.3389/fpsyg.2015.00295

**Published:** 2015-03-17

**Authors:** Julia F. Dietrich, Stefan Huber, Hans-Christoph Nuerk

**Affiliations:** ^1^Knowledge Media Research CenterTübingen, Germany; ^2^Department of Psychology, Eberhard Karls UniversityTübingen, Germany; ^3^LEAD Graduate School, Eberhard Karls UniversityTübingen, Germany

**Keywords:** approximate number system, measuring, tasks, reliability, validity, stimuli, visual control, presentation duration

## Abstract

According to a dominant view, the approximate number system (ANS) is the foundation of symbolic math abilities. Due to the importance of math abilities for education and career, a lot of research focuses on the investigation of the ANS and its relationship with math performance. However, the results are inconsistent. This might be caused by studies differing greatly regarding the operationalization of the ANS (i.e., tasks, dependent variables). Moreover, many methodological aspects vary from one study to the next. In the present review, we discuss commonly used ANS tasks and dependent variables regarding their theoretical foundation and psychometric features. We argue that the inconsistent findings concerning the relationship between ANS acuity and math performance may be partially explained by differences in reliability. Furthermore, this review summarizes methodological aspects of ANS tasks having important impacts on the results, including stimulus range, visual controls, presentation duration of the stimuli and feedback. Based on this review, we give methodological recommendations on how to assess the ANS most reliably and most validly. All important methodological aspects to be considered when designing an ANS task or comparing results of different studies are summarized in two practical checklists.

## Introduction

Between 2008 and 2013 the number of publications regarding the approximate number system (ANS) increased considerably. In this time period, a lot of research dealt with the question, whether the ANS is related to symbolic math abilities (see De Smedt et al., 2013; Feigenson et al., 2013; Chen and Li, 2014; Fazio et al., 2014 for meta-analyses and reviews). In our society today math abilities are essential for graduation and professional success as well as for psychological well-being, especially self-esteem (Bynner and Parsons, 2006). As the ANS is suggested as the basis for math abilities (e.g., Dehaene, 2001), the ANS might be thought of as the starting point for the acquisition of math abilities. Consequently, deficits in the ANS might lead to difficulties in the achievement of math abilities (Piazza, 2010; Piazza et al., 2010; Mazzocco et al., 2011). Hence, understanding the ANS and its relation to math abilities is important, as it offers the opportunity to identify children at risk of developing difficulties in math early on. In turn, this helps to prevent them falling farther and farther behind their class mates.

Furthermore, a good understanding of the causal factors for math difficulties enables the design of adequate interventions for children struggling with the acquisition of symbolic math abilities (see De Smedt et al., 2013 for an overview of educational interventions focusing on magnitude processing). Therefore, it was frequently investigated whether the ANS is a predictor of math abilities, but the findings so far are inconsistent (see De Smedt et al., 2013 for a review). However, many different approaches have been used to assess ANS acuity. In the present review, we give an overview of the approaches. Furthermore, we discuss the methods regarding psychometric features and summarize methodological aspects to be considered when measuring the ANS. As the methods employed derive from the theoretical concept ANS, we start by shortly introducing the ANS theory and typical characteristics of the ANS^1^.

## The ANS – Definition and Theory

The ANS is a cognitive system, which represents an imprecise estimate of the number of discrete entities in a set, i.e., the numerosity (Cantlon et al., 2009; Dehaene, 2009; see Stoianov and Zorzi, 2012, for a computational account of numerosity comparison). The ANS is assumed to support the comparison and estimation of numerosities as well as basic numerical operations, like approximate arithmetic (Dehaene, 2001, 2009).

**FIGURE 1:**
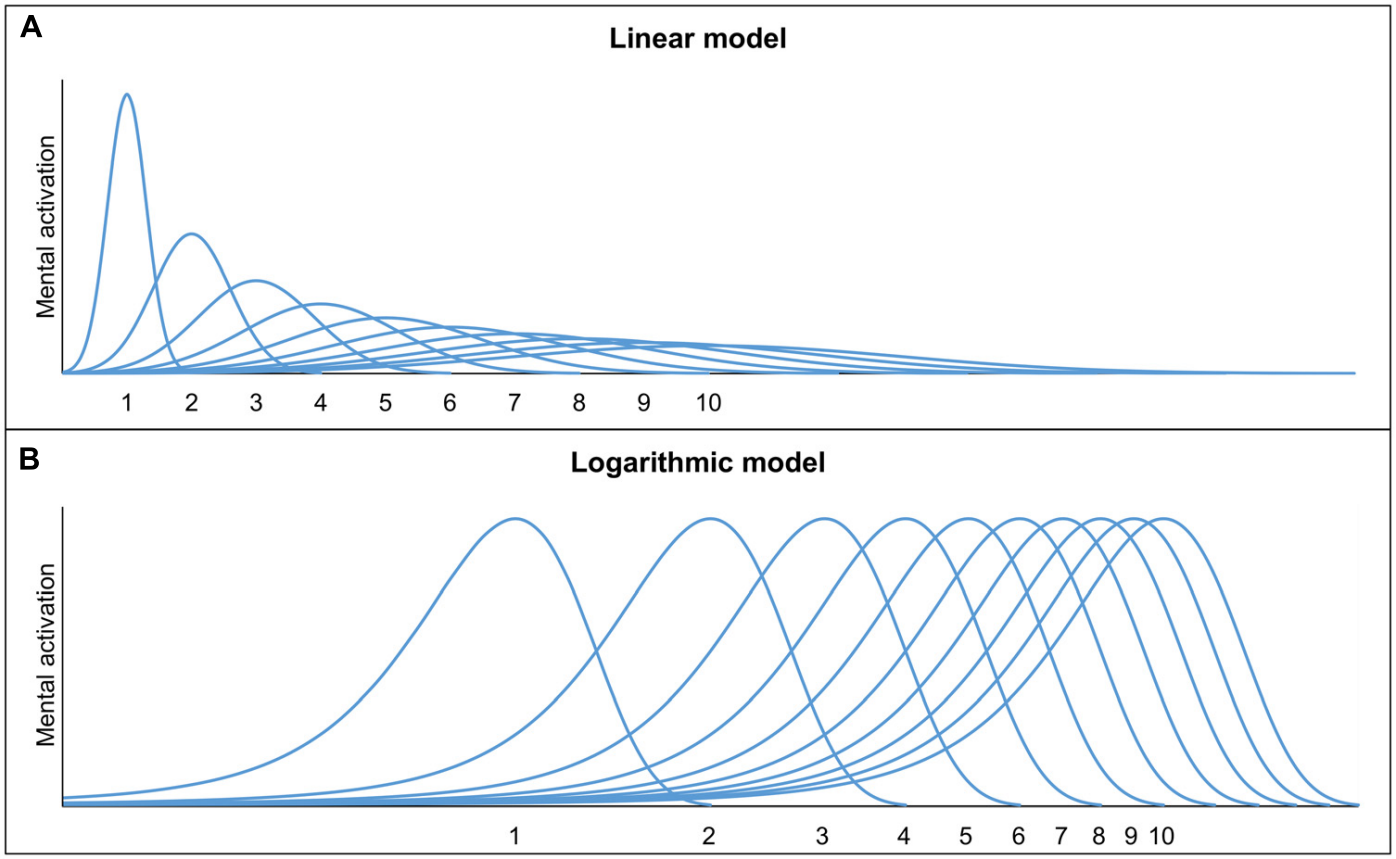
**Graphical illustrations of the linear model (A) and the logarithmic model (B)**.

**FIGURE 2:**
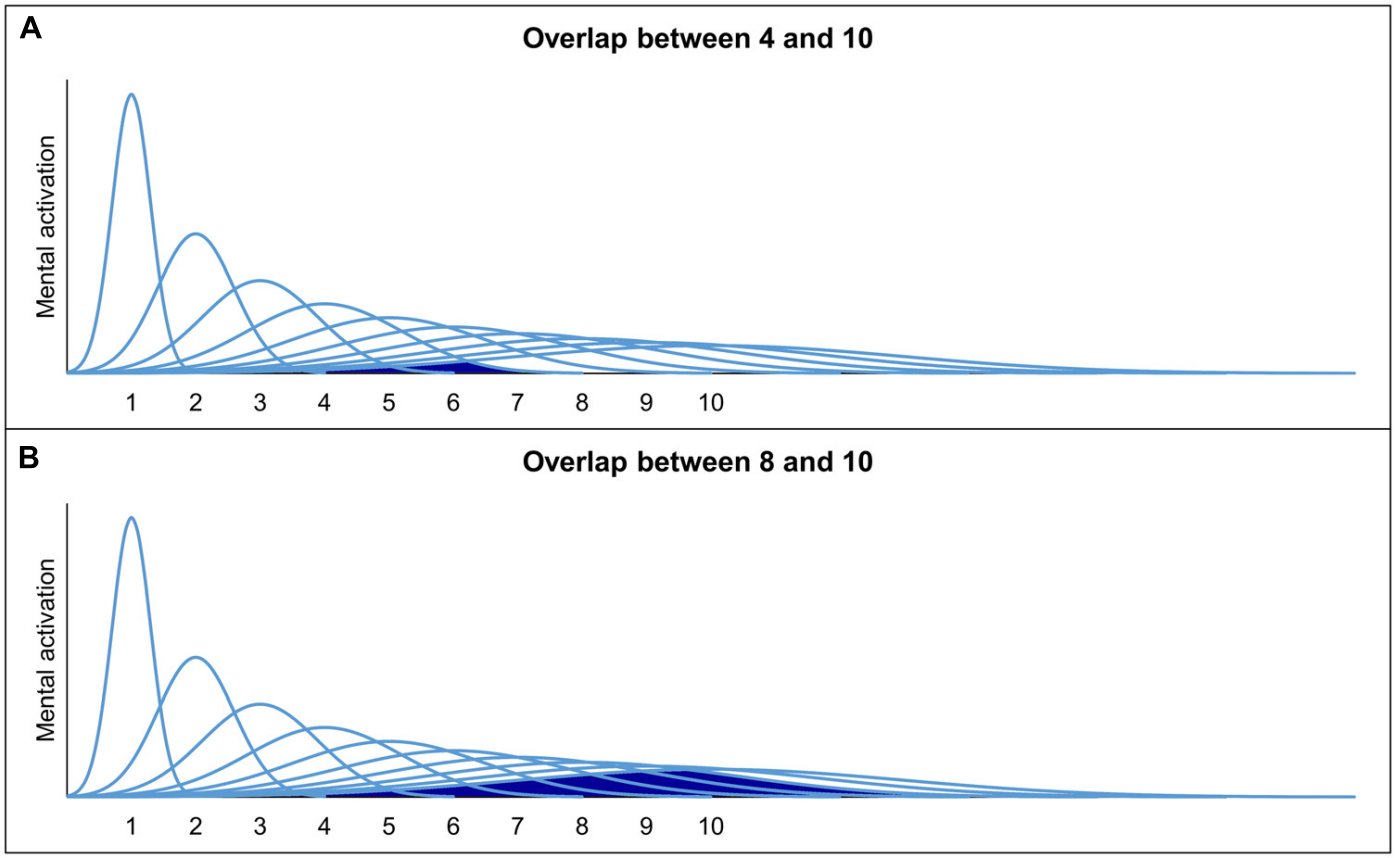
**Overlap between the ANS representations of 4 and 10 (A) as well as 8 and 10 (B) according to the linear model**.

The numerosities are assumed to be represented by overlapping Gaussian tuning curves. Each of the Gaussian tuning curves reflects the activity of neurons responding to specific numerosities, whereby the activation is at a maximum for one numerosity, but also adjacent numerosities cause activation (Feigenson et al., 2004). There are two models which describe the representation of non-symbolic quantities using Gaussian tuning curves (but see, e.g., Zorzi and Butterworth, 1999, for the alternative idea of summation coding): (1) the *linear model* with linear scaling and increasing variability (e.g., Gallistel and Gelman, 2000; Brannon et al., 2001) and (2) the *logarithmic model* with logarithmic scaling and fixed variability (e.g., Dehaene and Changeux, 1993; Dehaene, 2003). The two models are depicted in **Figure 1**. In both models, the Gaussian tuning curves overlap more and more, as the numerosities increase. Hence, both models make similar predictions (Feigenson et al., 2004). The increasing imprecision of the representations is an essential feature of the ANS, which results in a ratio-dependent performance. When comparing a set of 4 with a set of 10 entities (i.e., ratio 4:10 with ratio = smaller numerosity divided by larger numerosity), the overlap between the representations of 4 and 10 is assumed to be small and, hence, a good performance can be expected. In contrast, when comparing a set of 8 and a set of 10 entities (i.e., ratio 8:10) the overlap is larger which leads to a worse discrimination performance (see **Figure 2** for an illustration). Hence, discrimination performance is influenced by the ratio of the to-be-compared numerosities (i.e., ratio-dependent performance). The more the ratio between two sets approaches 1, the more difficult the comparison and accordingly, accuracy tends to reach chance level.

## Dependent Variables

Several dependent variables are used to index ANS acuity (i.e., the precision of the ANS representations reflected by the spread of the Gaussian functions). Beside general performance measures, like mean accuracy or response time, some measures assess consequences of the ANS representation and, therefore, can be used as dependent variables of the ANS acuity (e.g., the ratio or distance effect). Other measures reflect the precision of the ANS representations more directly (e.g., the Weber fraction).

### Mean Accuracy and Response Time

Following the idea that ANS acuity should also affect the performance in ANS tasks, mean accuracy and response time (aggregated over the employed ratios) are used to measure ANS acuity (Soltész et al., 2010; Lourenco et al., 2012; Kolkman et al., 2013). Using accuracy as ANS measure is based on the idea that the more precise the ANS representations are, the higher the accuracy in the task is in general. Furthermore, it is assumed that participants with more precise ANS representations respond faster in ANS tasks (De Smedt et al., 2013; but see Piazza et al., 2010).

### Numerical Ratio Effect (NRE) and Numerical Distance Effect (NDE)

The numerical ratio effect (NRE) is used as a measure of the ANS, because as a result of the overlapping ANS representations participants show ratio-dependent performance in non-symbolic comparison tasks. It describes the increase in response times and error rates depending on the ratio between two numerosities: the larger the ratio, the slower and less error-prone the responses. In short, the NRE measures the influence of the numerical ratio on task performance.

Similarly to the NRE, the numerical distance effect (NDE) reflects a typical pattern of results in non-symbolic comparison tasks that can be explained by the overlapping ANS representations and is, therefore, used as an ANS measure. The NDE refers to the increase in response time and error rates as the distance between the two to-be-compared numerosities increases (Buckley and Gillman, 1974). In the theoretical framework of the ANS, the better discrimination performance for more distant numerosities is explained by a smaller overlap in their Gaussian tuning curves. Both NRE and NDE are indicated by the size of the slope of a regression analysis with ratio or distance respectively as a predictor.

It is assumed that more accurate ANS representations are reflected in a smaller NRE and a smaller NDE (e.g., De Smedt et al., 2013). This means that participants with a more precise ANS acuity perform more similarly for both easier (i.e., small ratio, large distance) as well as more difficult (i.e., large ratio, small distance) items. In contrast, participants with a less precise ANS acuity are assumed to perform substantially worse in difficult items than in easy items. However, the interpretation that a smaller NRE/NDE reflects a better ANS acuity is problematic, as a smaller NRE/NDE might also be due to floor effects. For example, when participants have problems solving the task and, hence, their performance is close to the chance level, this would lead to similar accuracy rates for different ratios/distances independent of the size of the ratios/distances. As a consequence, the performance can be described by a flat slope which indicates a small NRE/NDE and, hence, might be misinterpreted as a good ANS acuity.

Due to the similarities between the NDE and the NRE it could be assumed that both measures are highly related and, therefore, can be used interchangebly. However, for a given distance, several ratios can be created so that both effects do not correlate (see **Table 1** for an example). Therefore, NDE and NRE might not be used interchangebly and results should not be compared directly. Furthermore, from a theoretical perspective the NRE might reflect the ANS representations better than the NDE, as in the latter the effect does not consider the magnitude of the numerosities and, therefore, cannot model the increasing imprecision of the ANS representations. In contrast, the magnitude of the numerosity affects the ratio between two numerosities and is, therefore, reflected in the NRE.

**Table 1:** Examples of two numerosities (*n1* and *n2*) showing different ratios, but identical distances.

*n1*	*n2*	Ratio	Distance
1	2	0.50	1
2	3	0.67	1
3	4	0.75	1
4	5	0.80	1
5	6	0.83	1
6	7	0.86	1


### Weber Fraction

The Weber fraction is often used as a measure of the ANS acuity, as the ANS is assumed to follow Weber’s Law (e.g., Libertus and Brannon, 2009; Libertus et al., 2011). According to Weber’s Law the discrimination threshold increases linearly with numerosity (Dehaene, 2003). Weber’s Law predicts that the ratio between two to-be-compared numerosities determines task performance, whereby task performance is better the more different two numerosities are relative to each other (Libertus and Brannon, 2009; Libertus et al., 2011). The Weber fraction for a dot comparison task can be estimated by using the following formula (with erfc being the complementary Gauss error function, e.g., Pica et al., 2004):

$$f_{acc}\left(n1,n2,w\right)=1-\frac{1}{2}erfc\left(\frac{\left|n_{1}-n_{2}\right|}{\sqrt{2w}\sqrt{n_{1}^{2}+n_{2}^{2}}}\right)$$

In this formula n1 and n2 denote the to-be-compared numerosities and w the Weber fraction. Note that there are other formulas to calculate the Weber fraction for approximate arithmetic tasks (see Pica et al., 2004) and habituation paradigms (see Piazza et al., 2004).

The Weber fraction indicates the width of the Gaussian tuning curves with a smaller (larger) Weber fraction indicating sharper (broader) Gaussian tuning curves. Therefore, the smaller the Weber fraction w is, the narrower the Gaussian curves are and the better the acuity of the ANS representations is (e.g., Inglis and Gilmore, 2014). Hence, the Weber fraction directly represents the ANS acuity. It has been suggested that another advantage of the Weber fraction is that it abstracts away details of exact ratio, distance, and magnitude of the numerosities used in a particular study. Hence, the Weber fraction might enable more meaningful comparisons across different studies. However, a recent study by Inglis and Gilmore (2014) challenged this assumption by showing that the Weber fraction depended on the ratios employed. Furthermore, the Weber fraction should not be calculated for participants with accuracy scores substantially below 50% (i.e., chance level), as this indicates that participants do not behave according to Weber’s Law (e.g., Inglis and Gilmore, 2014). Participants’ accuracy scores can be below 50%, when they rely on visual cues which are negatively correlated with numerosity causing systematic errors (see **Controlling Visual Properties**).

### Convergent Validity – Relationship between the Dependent Variables

All of these measures are assumed to index the ANS acuity and, therefore, should be correlated. However, there is first evidence that this is not the case. Price et al. (2012) and Inglis and Gilmore (2014) showed that correlations between ANS measures differ greatly. Only the measures accuracy and Weber fraction were strongly related (*r* = 0.89) and could be interpreted as presumably measuring the same construct (Inglis and Gilmore, 2014). The relationship between the NRE based on accuracy data (NRE_acc_) or the NRE based on response time data (NRE_RT_) and the accuracy measure was small (NRE_acc_: *r* = 0.14; NRE_RT_: *r* = 0.44) indicating a severe lack of convergent validity. Furthermore, the correlation between the NRE measures and Weber fraction was also low (NRE_acc_: *r* = 0.40; NRE_RT_: *r* = 0.37; Inglis and Gilmore, 2014). The latter result is consistent with the results of Price et al. (2012), who found only a small correlation between the NRE_RT_ and the Weber fraction (*r* = 0.33). According to Inglis and Gilmore (2014) this indicates that NRE and accuracy/Weber fraction do not measure the same construct. Interestingly, the NRE_acc_ and the NRE_RT_ were not correlated (*r* = 0.14) suggesting that the NRE_acc_ and the NRE_RT_ measure different constructs. Differences between the measures might be partially explained by differences between accuracy based measures (i.e., Weber fraction, mean accuracy, NDE_acc_/NRE_acc_) and RT based measures (i.e., mean RT, NDE_RT_/NRE_RT_). Inglis and Gilmore (2014) already pointed out that it is unclear whether ANS theory would predict a relationship between accuracy and RT based measures. Furthermore, Piazza et al. (2010) found significant differences between dyscalculics and controls regarding their ANS acuity measured with an accuracy based measure, but not for a RT based measure. In sum, most available results suggest that convergent construct validity over different dependent variables is rather poor.

### Reliability of the Dependent Variables

Furthermore, the different correlations between the ANS measures reported above might be explained by differences in reliability (see also Inglis and Gilmore, 2014). The reliability of a measure sets an upper bound on how high the correlation between two variables can be (Goodwin and Leech, 2006). Hence, the smaller the reliability of a measure, the smaller the potential size of the correlation. Thus, the small correlation between the NRE and the other ANS measures might be due to a low reliability of the measure NRE.

Inglis and Gilmore (2014) investigated the test–retest reliability of the ANS measures and found large differences between the measures regarding their reliabilities. Test–retest reliability of one week was largest for the accuracy measure (acc), followed by the Weber fraction (w), whereby the reliability was smaller for children (acc: *r* = 0.47; w: *r* = 0.41) than for adults (acc: *r* = 0.65; w: *r* = 0.60). In contrast, the NRE had a poor test–retest reliability, which did not reach significance. Again, the test–retest reliability was smaller for children (NRE_acc_: *r* = –0.13; NRE_RT_: *r* = –0.07) than for adults (NRE_acc_/NRE_RT_: *r* = 0.27). These results might also explain the small correlations between accuracy measure and the NRE or Weber fraction and the NRE, as the reliability of the NRE was very poor which in turn limited the size of the correlations (Goodwin and Leech, 2006). In contrast, as the reliabilities for accuracy measure and the Weber fraction were larger, the observable correlation between these measures was also larger.

Sasanguie et al. (2011) also reported a low split-half reliability for the NDE, both based on accuracy data and on response time data (NRE_acc_: *r* = 0.22; NRE_RT_: *r* = 0.40). According to Sasanguie et al. (2011), one potential causing factor of the low reliability for the NDE might be the approach of calculating difference scores, as reliability estimates were found to decrease when correlations were calculated using difference scores (e.g., Strauss et al., 2005). When calculating the reliability without using difference scores Sasanguie et al. (2011) found substantially larger correlations (*r* > 0.70).

Using unreliable measures has negative consequences for research, because it makes it difficult to detect correlations between two measures (e.g., between ANS acuity and math ability) or to detect group differences (e.g., differences between dyscalculia participants and control groups; Maloney et al., 2010). Reliability places an upper bound for the correlation between two variables (Goodwin and Leech, 2006). Therefore, differences in reliability might also explain different correlations between ANS acuity and math ability. Furthermore, a null effect might be caused solely by a lack of reliability of one of the measures and not by the absence of a relationship between the measures (Sasanguie et al., 2011). While satisfactory reliabilities have been obtained for adults at least in some studies, poor reliabilities seem to be a more serious problem for studies with children.

### Which ANS Measure should be Preferred?

Putting these results together, the NRE as well as the NDE are not recommended to be used when measuring the ANS acuity (Lindskog et al., 2013b; Inglis and Gilmore, 2014), as these measures show poor test–retest reliabilities and are not related with the other ANS measures (Sasanguie et al., 2011; Price et al., 2012; Inglis and Gilmore, 2014).

A good measure of the ANS acuity might be mean accuracy, as it combines good psychometric features (Inglis and Gilmore, 2014): firstly, the highest test–retest reliability of all ANS measures was reported for the accuracy measure. Secondly, accuracy measures were found to follow a normal distribution which is necessary when correlating the ANS acuity with other measures (e.g., math ability), because the Pearson correlation coefficient is sensitive to violations of the assumption of normality (Inglis and Gilmore, 2014). However, the distribution of the accuracy measure depends on the sample and the task difficulty. Hence, if the task is too simple or too difficult, this will result in ceiling or floor effects causing a skewed distribution. Therefore, the distribution of the ANS measures (regardless of whether accuracy measure, Weber fraction, or NDE/NRE were employed) has to be considered and checked, especially in correlation studies.

Nevertheless, the Weber fraction might also be a good measure, as Inglis and Gilmore (2014) reported similar reliabilities for the Weber fraction and the accuracy measure. Furthermore, both measures are highly correlated (Inglis and Gilmore, 2014). It is important that future studies report the same measures in order to allow researchers to accumulate evidence from several studies and to describe the development of ANS acuity in typical and atypical developing individuals.

So far, the relationship between mean RT and other measures of the ANS acuity has not been investigated systematically. However, there is evidence that RT based measures yield different results than accuracy based measures (Piazza et al., 2010). In general, it is important to check the reliability of measures before conducting correlation studies as it limits the size of correlations (Goodwin and Leech, 2006). Moreover, the distribution of the performance has to be inspected to exclude the possibility of ceiling or floor effects.

## ANS Tasks

Several different tasks allow the calculation of the aforementioned ANS measures. In all these tasks, two dot sets are presented which have to be compared or added/subtracted. The difficulty of the tasks depends on the ratio between the to-be-compared numerosities (De Smedt et al., 2013). The larger the ratio, the more difficult the discrimination is between the two sets. In research on the ANS, convergent validity across different ANS tasks is often implicitly assumed; i.e., that different tasks measure ANS acuity in the same manner (e.g., De Smedt et al., 2013; Feigenson et al., 2013). However, recent studies showing small or null correlations between different ANS tasks question this assumption (e.g., Price et al., 2012; Smets et al., 2014).

### Dot Comparison Tasks

The most commonly used task to measure the ANS acuity is the dot comparison task (e.g., De Smedt et al., 2013). In dot comparison tasks, two sets of dots are presented and participants have to determine which of the two sets contains more elements. There are several versions of the dot comparison task: (1) the paired task, where the to-be-compared sets are presented simultaneously, but separately on one screen, (2) the sequential task, where the two dot sets are presented successively, (3) the intermixed task, where the two sets consist of distinguishable colored dots (e.g., blue and yellow), which are presented intermixed on one screen.

Price et al. (2012) investigated the relationship between the three versions of the dot comparison task (intermixed, paired, and sequential). Using the Weber fraction as a measure of the ANS, they found significant correlations between all combinations of the task versions. However, the size of the correlations between the tasks varied by a substantial amount. The intermixed and the paired task correlated with *r* = 0.39, the intermixed and the sequential task with *r* = 0.68 and the paired and the sequential task with *r* = 0.50. Thus, the significant correlations indicate that the task versions appear to measure at least at some level the same concept, probably the ANS (e.g., De Smedt et al., 2013). Nevertheless, the significant correlations between the ANS tasks could also be due to general factors, like intelligence, attention, or motivation of the participants (e.g., Gebuis and Van der Smagt, 2011).

Furthermore, the differences regarding the correlations between the ANS task versions might be explained by different domain general cognitive demands of the tasks, like working memory or inhibitory control (e.g., Price et al., 2012; Gilmore et al., 2014). In the sequential task, the two sets of dots are presented successively. Therefore, it was suggested that this task variant requires additional working memory processes. In contrast, in the paired or in the intermixed task, the to-be-compared dot sets are presented on the same screen so that working memory processes should play a lesser role (Price et al., 2012; Gilmore et al., 2014). Furthermore, the intermixed task might demand additional processes, like visual resolution, to resolve the overlapping sets (Price et al., 2012; Gilmore et al., 2014). Nevertheless, these results raise the question as to how the construct of the ANS can be assessed in the most valid way.

#### Discriminant Validity – are We Really Measuring ANS Acuity?

One selection criterion for choosing among the ANS tasks is to use the “purest” task with the least involved additional cognitive processes. Hence, it could be recommended to employ the paired version, which appears to require the least additional cognitive processes and should therefore be preferred, in order to ensure that ANS acuity is measured rather than other domain general skills, like visual resolution, inhibitory control, or working memory (Price et al., 2012; Fuhs and McNeil, 2013; Gilmore et al., 2014).

Additionally, in studies correlating ANS acuity with math performance, it might be considered to control for domain general skills (e.g., working memory and inhibition) or intelligence in order to measure only unique variance of ANS acuity and math performance, as intelligence, inhibition, and working memory were found to correlate with math performance (Bull and Scerif, 2001; Deary et al., 2007; Friso-van den Bos et al., 2013).

Importantly, when measuring the ANS with a dot comparison task, a serious problem regarding discriminant validity is that the numerosity and visual properties of the stimuli are confounded (Gebuis and Reynvoet, 2011; Leibovich and Henik, 2013). This leads to the question, as to whether participants discriminate numerosity or rely on visual cues. To make sure that the ability to discriminate numerosities (i.e., the ANS) and not the ability to discriminate visual cues is studied, it is essential to control for the visual properties. There are several visual properties covarying with numerosity (Gebuis and Reynvoet, 2011). Usually the following visual properties are taken into account: (1) occupied area (i.e., the convex hull, which indicates the smallest contour consisting of all items), (2) cumulative surface area (i.e., the sum of all items surfaces), (3) item size (i.e., the average diameter of all items), (4) total circumference (i.e., the sum of the circumferences of all items), (5) the density of the items (i.e., the distance between the items, calculated by dividing area extended by total surfaces; Gebuis and Reynvoet, 2011). It is physically impossible to control for all properties at the same time. Controlling for one visual property leads to changes in another visual property (see Leibovich and Henik, 2013). There are several approaches to control for visual properties (see **Controlling Visual Properties**).

#### Reliability of the ANS Dot Comparison Tasks

Results from studies investigating the reliability of ANS tasks vary to a substantial degree. Gilmore et al. (2011, 2014) found good split-half reliabilities for the paired version of the dot comparison task, both for adult and children samples (*r* ≥ 0.85, based on accuracy data). Other studies, however, reported considerably lower reliabilities. Reliability coefficients higher 0.70 are commonly considered as acceptable. However, it depends on the measurement situation as to what constitutes adequate reliability (see Lance et al., 2006). In the study of Price et al. (2012), split-half reliabilities varied considerably depending on the employed variants of the dot comparison task between *r* = 0.44 for the sequential task, *r* = 0.47 for the paired task and *r* = 0.78 for the intermixed task (results based on the Weber fraction). When using the NRE as a measure of the ANS, the reliabilities varied between *r* = 0.57 for the intermixed task, *r* = 0.65 for the sequential task, and *r* = 0.78 for the paired task. Furthermore, Lindskog et al. (2013b) reported very low split-half reliabilities for the intermixed task version. For Weber fraction, accuracy measures and the NDE_RT_, they found split-half reliabilities of about 0.40, but a much lower reliability of 0.15 for the NDE_acc_. This finding is well in accordance with the study of Sasanguie et al. (2011) who found a larger split-half reliability for the NDE based on RT (*r* = 0.40) than for errors (*r* = 0.22) employing the paired task. In contrast, DeWind and Brannon (2012) reported good reliabilities for the intermixed task version: the spilt-half reliabilities ranged from 0.83 to 0.94. These differences in reliabilities might be explained by different number of trials or the variance in the sample employed to measure reliabilities.

As suggested by Lindskog et al. (2013b), a source for different reliabilities might be the number of trials employed. The more trials employed, the larger the reliability. However, this cannot explain differences in reliabilities in the study of Price et al. (2012), as the number of trials was held constant for the different task variants. Furthermore, Gilmore et al. (2014) employed the smallest number of trials, but reported the largest reliability.

Another explanation might be the between-subject variance of the sample employed to measure reliabilities (Goodwin and Leech, 2006), which has not been considered yet. The reliability depends on the variance of the performance in the task: the larger the variance, the better the reliability. For example, in the study of Price et al. (2012) the variance of the Weber fraction was greater in the intermixed task (*SD* = 0.13) than in the paired (*SD* = 0.04) or the sequential task (*SD* = 0.06), where the task performance was more similar. The larger variance in the intermixed task might therefore account for the larger reliability for this task variant. Differences in the variance of the performance in the three task variants might also explain differences in reliabilities for the NRE. For the NRE, Price et al. (2012) found the largest split-half reliability and also the largest variance between participants (*SD* = 164.92) in the paired task. Moreover, there was more variance in the sequential task (*SD* = 144.95) than in the intermixed task (*SD* = 121.28). Consistently, the split-half reliability was larger in the sequential task than in the intermixed task.

Therefore, future studies investigating reliability should consider the variability of task performance. However, sufficient variance is necessary not only for reliability studies, but also for correlation studies in general, as a small variance can result in an underestimation of the relationship (Goodwin and Leech, 2006). Hence, it is important to check the variance of the task performance of the participants, for example with a scatter plot, to ensure that the sample is not too homogenous and to exclude the possibility of ceiling and floor effects, which in turn result in a small variance. Finally, the results demonstrate the need for a standardized ANS task with good psychometric characteristics.

### Other Tasks

Beside the three dot comparison task versions, other tasks were also employed to measure ANS acuity, including the same-different task and approximate arithmetic tasks. Additionally, tasks employed in developmental psychology studies are discussed.

#### Same-Different Task

In the same-different task participants have to indicate whether the number of two simultaneously presented dot sets are numerically the same or different (e.g., Sasanguie et al., 2011). According to Sasanguie et al. (2011) the same-different task has an acceptable split-half reliability (*r* = 0.65).

Smets et al. (2014) compared the performance of the same participants in a paired version of the dot comparison task and a same-different task. They found ratio-dependent performance for both tasks which is characteristic for the ANS and might suggest that the tasks measure the ANS. However, the tasks did not correlate significantly, neither when correlating accuracy scores (*r* = 0.15) nor when correlating Weber fraction (*r* = –0.28). These results could not be explained by a lack of reliability, as this possibility was ruled out by the authors, who found sufficient split-half reliability scores (*r* > 0.88). However, the performance in both tasks differed substantially. Performance in the comparison tasks was quite good with a mean accuracy of 79%, whereas the performance in the same-different task was close to chance level (i.e., 50%) with a mean accuracy of 60%. This suggests that participants might have guessed in a lot of trials when solving the same-different task, whereas in the dot comparison task they were able to select the larger numerosity in many more trials possibly measuring the ANS acuity. Hence, these performance differences might explain why the correlation between the same-different task and the dot comparison was not significant in the study of Smets et al. (2014).

In contrast to the results of Smets et al. (2014), Sasanguie et al. (2011) found that the NDE based on a paired dot comparison task and a same-different task were significantly correlated (*r* = 0.52). Moreover, Smets et al. (2013) found that the NDE did not significantly differ between a paired dot comparison task and a same-different task. In addition, Gebuis and Van der Smagt (2011) found the overall performance (i.e., accuracy measure) in a sequential version of the dot comparison task to be significantly correlated with a same-different task (*r* = 0.78). The latter results support convergent validity – at least for the paired or the sequential dot comparison task and the same-different task – suggesting that the tasks capture the same construct, probably the ANS acuity.

Taken together, there is evidence that the same-different task is related to the dot comparison task variants sequential and paired. The deviant results of Smets et al. (2014) might be due to the more difficult same-different task and the resulting low task performance. In the studies of Gebuis and Van der Smagt (2011) and Sasanguie et al. (2011), who found significant correlations between the dot comparison task and the same-different task, the performance in the same-different task differed significantly from chance level. These results show that it is also important to consider the performance of the participants at an absolute level.

#### Approximate Arithmetic Task

In addition to these dot comparison tasks, the ANS acuity can also be studied using approximate arithmetic tasks (e.g., McCrink et al., 2007; McCrink and Spelke, 2010, see also Knops et al., 2014). In the approximate arithmetic task, three dot sets are presented successively. Participants are asked to add or subtract the second dot set to/from the first one and compare the result with the third dot set. Gilmore et al. (2011, 2014) found good split-half reliabilities for non-symbolic addition tasks, both for adult (*r* = 0.93) and children samples (*r* = 0.87). Furthermore, the approximate arithmetic task seems to assess at least to some extent the same construct as the paired dot comparison task. However, a significant correlation between both tasks was found only in children (*r* = 0.43) and not in adults (acc: *r* = 0.04, w: *r* = –0.01; Gilmore et al., 2014). Gilmore et al. (2014) suggested that the differences between children and adults might be due to differences regarding the extent to which task performance reflects ANS acuity or general cognitive processes.

### Deciding on an ANS Task

Taken together, the commonly used paired version of the dot comparison task appears to be the task of choice when measuring the ANS acuity both in children and in adults, as it requires less additional cognitive processes than the sequential dot comparison task, the intermixed dot comparison task or the approximate arithmetic task. Furthermore, the paired dot comparison task might be preferred to the same-different task, as the latter was found to be more difficult than the dot comparison task, which increases the risk of floor effects. This is a problem for correlation studies, as due to the reduced variance of the task performance the relationship might be underestimated (Goodwin and Leech, 2006). For correlation studies it is also important that the task has a good reliability. For the paired dot comparison task, studies showed mostly acceptable reliabilities (Gilmore et al., 2011, 2014; Price et al., 2012). Nevertheless, all tasks used to assess the ANS acuity were correlated in most of the studies indicating that they measure at least to some extent the same construct.

### Measuring the ANS in Young Children and Infants

Studying the ANS in young children and infants requires different experimental procedures and, in the case of infants, also different tasks. When measuring the ANS in young children (i.e., at an age of 3 or 4 years) it is important to make sure that the children really understand the task. When children do not understand the instruction “where are more dots?,” their responses might rely solely on guessing or they might misinterpret the instruction and compare visual dimensions, like the size of the dots (Negen and Sarnecka, 2014). To avoid such methodological artifacts and make sure that ANS is measured, it is recommended to ensure that the children understand the task and compare the numerosity of the dot sets to be compared. According to Negen and Sarnecka (2014), this can be achieved by employing a set of training trials with an easy ratio (1:3) and detailed feedback explaining errors, for example, “those dots are bigger, but this side has more dots, they’re smaller, but there are more of them” (Negen and Sarnecka, 2014). Only after several training trials have been solved correctly should the actual test trials be employed. Such a procedure ensures that the task really measures the ANS and, hence, contributes to a valid assessment of ANS acuity.

To investigate the ANS and its development in infants, other paradigms than the dot comparison tasks are required, because infants cannot perform tasks requiring verbal instructions. The most common paradigms are the habituation paradigm (e.g., Xu and Spelke, 2000) and the preferential looking paradigm (Libertus and Brannon, 2010).

In habituation paradigms, a set of dots with a specific numerosity is presented in each trial. From trial to trial the position of the dots as well as the dot size varies, but the number of dots stays constant. Infants’ looking time on the stimuli declines the more often the same numerosity is presented (i.e., they habituate to the stimulus). After the children habituate to the numerosity, a target stimulus is presented which is a set of dots with a different numerosity. When the infants notice the change in numerosity, this can be seen in a longer looking time (Gebuis and Van der Smagt, 2011).

In the preferential looking paradigm infants are shown two streams of dot patterns, one stream remains constant in numerosity, whereas in the other stream two numerosities are alternated. Infants prefer looking at the numerically changing stream, whereby looking times vary as a function of the ratio.

Using such paradigms, it was shown that infants at the age of 6 months can already discriminate numerosities with a ratio of 1:2, while 10 month-olds already discriminate ratios of 2:3 (Xu and Spelke, 2000; Xu and Arriaga, 2007). These results from developmental psychological paradigms were compared with results of dot comparison tasks (e.g., Halberda and Feigenson, 2008). Using a dot comparison task, Halberda and Feigenson (2008) found that between the ages of 3 and 6 years, the discrimination performance increases from a ratio of 3:4 to a ratio of 5:6. Finally, adults can discriminate even a 10:11 ratio (Halberda and Feigenson, 2008; see Piazza et al., 2010 for similar results). These results are commonly interpreted to imply that the ANS representations of infants are more imprecise than those of children and adults and that the discrimination ability of numerosities improves continuously with age (e.g., Feigenson et al., 2013). However, this conclusion is only justified, when these tasks measure the same construct and are comparable regarding the difficulty of the tasks. Using a non-symbolic dot comparison task, Starr et al. (2013) found that the ANS acuity of 3 month-old infants measured with a preferential looking paradigm predicted their ANS acuity at 3.5 year-olds. This result provides evidence that both tasks measure – at least to some extent – the same construct. However, several studies found that the tasks used in infants and in adults differ in their difficulty.

Gebuis and Van der Smagt (2011) compared the task performance in a dot comparison task with an explicit version of a habituation task (i.e., change detection task). In the change detection task a stream of dot sets was presented with occasionally changing numerosities. Participants had to indicate when they detected a numerically deviant dot set. The performance in the detection task was significantly worse than in the dot comparison task (Gebuis and Van der Smagt, 2011). Similar results were found in the study of Smets et al. (2014) who created another version of the change detection task following the preferential looking paradigm. In this change detection task version, two parallel streams of dot sets were presented, whereby in one stream the numerosities remained constant, while in the other stream two numerosities alternated. Participants had to indicate the stream that changed in numerosity. In line with the results of Gebuis and Van der Smagt (2011), Smets et al. (2014) found a significantly worse performance in the change detection task than in the comparison task.

These results question conclusions about the developmental changes in the ANS acuity. When the tasks employed in infants are more difficult than the dot comparison task used in older children or adults, the worse discrimination performance in children might not be due to more imprecise ANS representations but solely due to the more difficult task employed (Gebuis and Van der Smagt, 2011). However, Halberda and Feigenson (2008) also found differences between the age groups 3-, 4-, 5-, and 6- year-olds, although all age groups conducted a dot comparison task. Thus, the latter results suggest that at least from the age of 3, the ANS representations appear to become increasingly more precise (see also Piazza et al., 2010).

Taken together, when investigating the ANS acuity in children, it has to be checked whether they understand the ANS task. Furthermore, when investigating the ANS acuity in infants, developmental psychological paradigms, like habituation, are required. Nevertheless, it is important to use similar tasks for infants, children and adults, wherever possible, to ensure that the results of different age groups are comparable and reflect changes in ANS acuity, not in the difficulty of the tasks. However, so far it is not clear which tasks can be used subsequently to the habituation or preferential looking paradigm. The change detection task was constructed in analogy to these two paradigms and, therefore, it could be assumed that the results of the change detection task can be compared to the results of the developmental psychology paradigms. However, Gebuis and Van der Smagt (2011) already questioned whether the results of passive viewing, as in habituation paradigms, and active comparison, as in the change detection task, can be compared. One possible alternative approach might be fMRI adaptation paradigms allowing for passive processing of the stimuli both in infants and in older age groups (e.g., Piazza et al., 2007). Hence, further studies are needed to develop tasks in order to be able to investigate changes in the ANS acuity over the course of development.

## Methodological Aspects Relevant for Measuring ANS

We have already reviewed several different tasks used to measure the ANS and several measures employed to index ANS acuity, which cannot be used interchangeably. A summary of their psychometric features and relevant methodological problems is given in **Table 2**. However, there are many more methodological aspects, which vary greatly from one ANS study to the next and could contribute to inconsistent findings. Especially studies investigating the relationship between ANS acuity and math performance differ greatly regarding the design of the ANS task (De Smedt et al., 2013; Feigenson et al., 2013). Hence, differences between the studies are hard to interpret as studies differ in regard to many different aspects. The following section gives an overview of the most relevant aspects and can serve as a checklist when measuring the ANS or when comparing the results of different studies.

**Table 2:** Checklist of psychometric features and methodological problems regarding dependent variables and tasks of the ANS.

Methodological problem	Recommendation	Reason
**Reliability**: not all **dependent variables** are reliable.	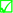 Use accuracy 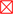 Do not use NRE or NDE	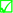 Best reliability. 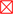 Poor reliability (Sasanguie et al., 2011; Inglis and Gilmore, 2014). *Note*: Reliability depends on age and is smaller for children.
**Convergent validity**: many **dependent variables** are not correlated.	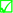 Use accuracy or Weber fraction 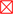 Do not use NRE	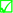 Only accuracy and Weber fraction were strongly related and measure presumably the same construct. 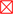 The NRE was not related with other dependent variables of the ANS (Inglis and Gilmore, 2014).
**Sample distribution**: ceiling/floor effects lead to skewed distributions.	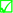 Check distribution of the dependent variables using a scatter plot	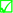 Many statistic procedures (e.g., Pearson correlation coefficient) assume normality distribution; skewed distributions violate this assumption (Inglis and Gilmore, 2014).
**Reliability**: not all **ANS tasks** are reliable.	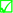 Use paired dot comparison or the approximate addition task	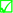 Reliabilities of both measures are (mostly) acceptable (e.g., Gilmore et al., 2011; Price et al., 2012).
**Convergent validity**: not all **ANS tasks** are correlated.	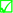 Use paired dot comparison task	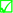 The paired dot comparison task is best studied and correlates with almost all other ANS tasks (e.g., Sasanguie et al., 2011; Price et al., 2012).
**Discriminant validity**: the **ANS tasks** should measure the ANS and no other cognitive processes.	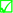 Use paired dot comparison or the same-different task 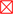 Do not use sequential/intermixed comparison or approximate arithmetic	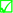 These two tasks can be considered as the “purest” ANS tasks with least involved additional cognitive processes. 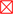 All tasks require additional cognitive processes, like working memory, or visual resolution (e.g., Price et al., 2012).
**Measuring ANS in young children:** children have to understand the instructions.	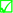 Start with training trials and detailed feedback 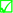 Check task understanding	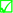 When children do not understand the task, they might just guess or compare visual properties (Negen and Sarnecka, 2014).
**Measuring developmental changes:** different tasks, varying regarding difficulty, are used in infants, and children/adult.	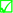 Use similar tasks for all age groups (e.g., change detection task, fMRI adaptation paradigm)	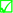 Tasks used to measure ANS acuity in infants (e.g., habituation paradigms) are more difficult than tasks employed in children/adults (Gebuis and Van der Smagt, 2011). Thus, the use of different tasks with varying difficulties can bias developmental changes.


### Stimuli

Regardless of which ANS task is employed, there are many aspects regarding the design of the stimuli which could influence the results, including the range of the stimuli, the ratios between the stimuli and, finally, the visual properties of the stimuli.

#### Stimulus Range

The stimulus range differs considerably from one study to the next. Some studies focused on small numerosities in the range of 1–9 elements (e.g., Mundy and Gilmore, 2009). Others used a larger range of numerosities ranging from 9 to 70 (e.g., Inglis et al., 2011). Hence, some studies also included numerosities within the subitizing range. Subitizing denotes the ability to recognize numerosities ranging from 1 to 3 entities immediately and accurately (Mandler and Shebo, 1982; Trick and Pylyshyn, 1994). Several studies indicate that two different systems exist for the processing of numerosities: the subitizing system for the processing of numerosities up to four and the ANS for numerosities larger than 4 (e.g., Feigenson et al., 2004; Revkin et al., 2008; Cutini et al., 2014).

Thus, to make sure that only the ANS is measured and no other process, like subitizing, we recommend not including the subitizing range in ANS studies. Furthermore, stimuli just above subitizing range can be counted easily. To ensure that the numerosities are estimated based on the ANS and are not solely counted, it might be advisable to avoid small numerosities.

#### Ratios – Manipulating Difficulty

Studies also differ greatly regarding the employed ratios, and, therefore, in the difficulty of the task. For example, in the study of Holloway and Ansari (2009) the easiest trial was to compare 1 dot with 9 dots (i.e., a ratio of 0.11). In contrast, in the study of Castronovo and Göbel (2012), the smallest ratio was much more difficult, because 12 and 16 dots (i.e., a ratio of 0.75) had to be compared. The more difficult the items – especially the easiest item in the task – the more likely floor effects might be. Similarly, the items employed to assess the upper discrimination ability should not be too simple, as this would in turn lead to ceiling effects. To avoid ceiling or floor effects, it is advisable to employ a wide range of different ratios.

However, it has to be kept in mind that the difficulty of a task is not solely reflected by the ratio between the employed stimuli. As mentioned above, same-different tasks are more difficult than dot comparison tasks. Hence, the performance on the same ratio can vary substantially depending on the employed ANS task (e.g., see Figure 3 in Smets et al., 2014). Furthermore, different age groups vary regarding their performance on the same ratios (Halberda and Feigenson, 2008). Therefore, the factors ANS task and age of the participants have to be considered, when deciding on the range of ratios optimal for a given age or task. When stimulus selection leads to a too high or too low difficulty, correlations such as reliabilities or validities will probably be underestimated, because there can be no covariance when there is no variance (Goodwin and Leech, 2006). Hence, we recommend avoiding too easy and too difficult ANS tasks which result in either ceiling or floor effects.

#### Controlling Visual Properties

Controlling visual properties is essential to ensure that the task really measures the ability to discriminate numerosity and not the ability to discriminate visual cues (see **Discriminant Validity – are We Really Measuring ANS Acuity**). There are several approaches to control visual properties and studies differ greatly regarding their designs to control for visual properties. Some studies controlled only one visual property at a time (e.g., Libertus et al., 2007; Bartelet et al., 2014). However, controlling for a single visual property is not sufficient, as according to Gebuis and Reynvoet (2011) participants would be likely to switch between reliable visual properties and not compare numerosities. Gebuis and Reynvoet (2011) developed a program to generate non-symbolic stimuli for which the visual properties explain only a small proportion of the variance of the distance between the numerosities. This program also provides information about the visual properties of the stimuli so that it is possible to analyze *post hoc* whether visual properties are confounded with distance between the numerosities. Thus, this program enables researchers to control for the possible confound of the distance between the numerosities and visual properties over all items employed in an experiment.

Another method is to keep visual properties negatively correlated to numerosity so that the visual properties provide opposing information to numerosity (Szücs et al., 2013). However, hereby not (only) the precision of the ANS representations might be measured, but also inhibitory control (Gilmore et al., 2013). In case of incongruent items, the visual and the numerical information is conflicting. Hence, participants have to inhibit the response to the visual information and respond solely to the numerosity (Gilmore et al., 2013; Szücs et al., 2013). Furthermore, Fuhs and McNeil (2013) found that inhibitory control correlated significantly with the performance in tasks, where numerosity and surface area were inversely related (*r* = 0.31). When equating mean surface area across trials such that surface area and numerosity were correlated, task performance was only marginally significant correlated with inhibitory control (*r* = 0.20). These results support the notion that other processes are involved in dot comparison tasks (see also Cappelletti et al., 2014). This result is important for correlation studies which investigate the relationship between ANS acuity and math performance, as inhibitory control is known to correlate with math performance (e.g., Bull and Scerif, 2001; St Clair-Thompson and Gathercole, 2006). Hence, significant correlations between ANS acuity and math performance might also be explained by both tasks measuring inhibitory control. In order to ensure that correlations with math performance are not mediated by inhibitory control it is important to control for this variable.

To sum up, the confounds between numerosity and visual properties are one of the biggest challenges in the research of the ANS, as the ANS was suggested to be an abstract representation of numerosity independent of perceptual variables (Lindskog et al., 2013b). Therefore, it is important to control the visual properties as stringently as possible and – because it is not possible to perfectly control for the visual properties – it is recommended to register them like Gebuis and Reynvoet (2011) in order to check possible confounds via *post hoc* analyses. Moreover, inhibitory control should be measured using another task and controlled for in correlation studies.

Moreover, research on numerosity estimation also addressed the problem of confounding visual cues and numerosity and proposed a novel method based on second-order (contrast-based) visual motion (Kramer et al., 2011). This methodology could be proficiently used for numerosity comparison tasks in order to prevent participants from using visual cues.

### Design

Besides these aspects related to the selection of stimuli, there are other factors concerning the design of the ANS task, which have an impact on the task performance.

#### Presentation Duration

The presentation duration is strongly linked to the performance in an ANS task. Inglis and Gilmore (2013) found that the performance increased with the presentation duration of the stimuli. Authors have often argued for the use of short presentation durations in order to avoid participants using counting strategies and to ensure that participants’ performance reflects the acuity of the ANS representations and not strategies (e.g., Halberda et al., 2008; Inglis et al., 2011). Moreover, using counting strategies could result in a more accurate performance than when relying solely on the imprecise ANS and, therefore, increase the risk of ceiling effects. However, there is no consensus as to how long the stimuli have to be presented to prevent counting strategies. For example, Halberda et al. (2008) presented the stimuli for 200 ms to ensure participants were not counting. Also Inglis et al. (2011) chose to restrict presentation duration to prevent participants from counting. However, they presented the stimuli for 1,500 ms in a child sample and limited the presentation to 1,249 ms in an adult sample.

The longer the presentation duration, the more likely counting strategies are. Therefore, presenting stimuli only for a short time might be advisable. Inglis and Gilmore (2013) found that participants were able to compare stimuli which were presented for only 16 ms, suggesting that the processing via ANS is automatic and/or extremely fast (Inglis and Gilmore, 2013)^2^. Thus, presentation durations around 100 ms should be long enough to measure the ANS, but also short enough to prevent participants from using counting strategies.

Other approaches to prevent counting strategies are instructing participants explicitly not to count (e.g., Bartelet et al., 2014) and/or interrupting when they are counting (Bonny and Lourenco, 2013) or show signs of counting, like pointing to the dots (Fuhs and McNeil, 2013).

To sum up, discrimination performance in dot comparison tasks is affected by the presentation duration of the stimuli. In order to ensure that participants rely on their ANS and not on counting strategies, it is recommended to restrict the presentation duration. Additionally, it can be helpful to explicitly instruct participants not to count and interrupt them when they obviously use counting strategies.

#### Feedback

Several studies investigated effects of feedback and training in ANS tasks (e.g., DeWind and Brannon, 2012; Lindskog et al., 2013a; Park and Brannon, 2013; Hyde et al., 2014). DeWind and Brannon (2012) found that the ANS acuity of participants improved when trial-by-trial feedback was employed. Although Lindskog et al. (2013a) could not replicate the results of DeWind and Brannon (2012), they found that the performance in the ANS task was marginally better in the feedback group than in the control group without feedback. Their results further suggest that feedback can have motivational effects. Moreover, it can also help participants to improve or shift their strategies, like using perceptual cues or counting strategies (Lindskog et al., 2013a). Thus, to study the unbiased ANS acuity, feedback should be avoided to make sure that no additional processes, like the adaptation of strategies or changes of the ANS acuity, influence the results. However, feedback is useful when using training to investigate how the ANS can be improved.

#### Number of Trials

Another important aspect when developing an experimental task is to determine the number of trials of the experiment. The reliability of ANS tasks depends on the number of trials: it increases monotonically as a function of the number of trials (Lindskog et al., 2013b). So far studies investigating the ANS acuity differ substantially regarding the number of trials employed. There are studies employing less than 50 trials (e.g., Fuhs and McNeil, 2013) and studies with more than 600 trials (e.g., DeWind and Brannon, 2012). According to the results of Lindskog et al. (2013b), the split-half reliability of intermixed comparison tasks with 50–200 trials is quite low, with reliability coefficients below 0.5. An acceptable reliability of 0.7 can be reached by employing about 400 trials. One possibility to reduce the number of trials is using adaptive procedures (Lindskog et al., 2013b). However, Lindskog et al. (2013b) focused only on an intermixed comparison task. For the other ANS tasks, the influence of the number of trials on reliability has not yet been investigated. In any case, it is important to check the reliability when conducting a correlation study.

To date, no study has yet investigated how many trials are required per ratio to get an accurate estimate of the Weber fraction or the NRE. For the Weber fraction, an estimate can be calculated taking into account the results of Lindskog et al. (2013b). They found an acceptable reliability when employing a total of 400 trials. As they used five different ratios, 80 trials per ratio might result in an acceptable reliability. However, further studies are required which systematically investigate the number of trials needed to obtain an acceptable reliability.

## Conclusion

The ANS is argued to be the foundation of symbolic math abilities (Dehaene, 2001). Therefore, the relationship between ANS acuity and math abilities was studied extensively, but findings are inconsistent (De Smedt et al., 2013). In this review, we summarized evidence suggesting that discrepancies in findings about the relationship between ANS acuity and math abilities might be partially explained by differences in the reliability of ANS measures. This is due to the fact that the dependent variables of ANS acuity vary substantially regarding their reliability, limiting the size of the correlation between ANS acuity and math abilities. Thus, the relationship between ANS acuity and math performance should be greater for measures with a larger reliability, like mean accuracy, than for measures with a lower reliability, like the NDE. In line with this suggestion, a recent meta-analysis by Chen and Li (2014) found larger correlations between ANS acuity and math abilities for mean accuracy than for the NDE.

Furthermore, this review gave an overview of relevant methodological aspects in the measurement of the ANS. Based on the existing evidence, recommendations for measuring the ANS in future studies were given, which can be summarized as follows: when investigating developmental changes in the ANS acuity, it is important to use a similar task for infants, children and adults. For children or adults a paired version of the dot comparison task could be recommended, because it has good psychometric features, i.e., reliability and construct validity. Regarding the measures of the ANS, the accuracy measure and the Weber fraction seem to be the measures of choice, while the NDE or NRE cannot be recommended based on the current literature. Independently of the tasks and the measures used to assess ANS acuity, it is important to check whether participants can solve the task and how the task performance varies, as too homogenous samples limit the size of correlations. In addition, there are several other aspects to be considered when designing an ANS task or comparing the results of different ANS tasks. We provide a practical checklist for researchers which summarizes all relevant aspects to be considered and provides recommended solutions for methodological problems based on the available literature (see **Tables 2** and **3**).

**Table 3:** Checklist of methodological aspects relevant for measuring the ANS.

Methodological aspect	Recommendation	Reason
Stimulus range	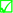 Use numerosities beyond subitizing range (i.e., larger 4)	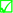 Numerosities up to four are processed via subitizing and, therefore, not based on the ANS (Feigenson et al., 2004). Furthermore, also stimuli just above subitizing range might be counted easily and should, therefore, be avoided.
Presentation duration	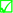 Use short presentation durations (around 100 ms)	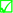 Inglis and Gilmore (2013) found that 16 ms were sufficient to compare two dot sets. 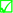 Additionally, counting strategies are prevented.
Ratios	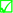 Use a wide range of different ratios	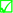 Too difficult/too simple tasks can result in floor/ceiling effects *Note*: difficulty of tasks does not solely depend on ratios, but also on age or the task employed (e.g., same-different task is more difficult than dot comparison tasks, e.g., Smets et al., 2014).
Visual confounds	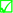 Control for visual cues	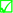 Controlling for visual cues is necessary to ensure that numerosity is processed and not visual cues (Gebuis and Reynvoet, 2011).
Feedback	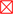 Avoid feedback	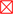 Feedback affects motivation, might enhance strategy use or ANS acuity (DeWind and Brannon, 2012; Lindskog et al., 2013a).
Number of trials	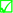 Employ about 400 trials 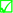 Use an adaptive procedure	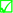 To reach acceptable reliability about 400 trials are necessary. 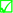 An adaptive procedure is more economic (Lindskog et al., 2013a).


## Conflict of Interest Statement

The authors declare that the research was conducted in the absence of any commercial or financial relationships that could be construed as a potential conflict of interest.

## Footnotes

1 In this review, we focused on non-symbolic stimuli as it is debated whether a unitary representation for symbolic and non-symbolic stimuli exists (for a review, see e.g., Cohen Kadosh and Walsh, 2009). While the relationship between non-symbolic magnitude and symbolic magnitude is an important (and also controversial) issue, it is beyond the scope of this paper.

2 Furthermore, the authors found that also within the range of presentation durations that prevent participants from counting, discrimination performance improved the longer the stimuli were presented, suggesting that besides counting there are other mechanisms influencing performance (see Inglis and Gilmore, 2013 for a detailed discussion).
